# Antenatal and postnatal cervical precancer screening to increase coverage: experience from Battor, Ghana

**DOI:** 10.3332/ecancer.2023.1616

**Published:** 2023-10-30

**Authors:** Kofi Effah, Ethel Tekpor, Gifty Belinda Klutsey, Hannah Tiwaah Bannor, Joseph Emmanuel Amuah, Comfort Mawusi Wormenor, Seyram Kemawor, Stephen Danyo, Bernard Hayford Atuguba, Lawrencia Serwaa Manu, Nana Owusu Mensah Essel, Patrick Kafui Akakpo

**Affiliations:** 1 Catholic Hospital, Battor, PO Box 2, Battor, via Sogakope, Volta Region, Ghana; 2 School of Epidemiology and Public Health, Faculty of Medicine, University of Ottawa, 451 Smyth Road (2046), Ottawa, ON K1H 8M5, Canada; 3 Department of Emergency Medicine, College of Health Sciences, Faculty of Medicine and Dentistry, University of Alberta, 730 University Terrace, Edmonton, AB T6G 2T4, Canada; 4 Department of Pathology, Clinical Teaching Center, School of Medical Sciences, University of Cape Coast, Cape Coast, Ghana; a https://orcid.org/0000-0003-1216-2296; b https://orcid.org/0000-0001-5494-5411; c https://orcid.org/0000-0003-0356-0663

**Keywords:** uterine cervical neoplasm, prenatal care, postnatal care, human papillomavirus infection, colposcopy, human papillomavirus DNA testing

## Abstract

**Background:**

Cervical precancer screening in low-resource settings is largely opportunistic with low coverage. Many women in these settings, where the burden of cervical cancer is highest, only visit health institutions when pregnant or after delivery. We explored screening during antenatal and postnatal visits aimed at increasing coverage.

**Methods:**

Pregnant women (in any trimester) attending antenatal care (ANC) and women attending postnatal care (PNC; 6–10 weeks) clinics were screened at Catholic Hospital, Battor and at outreach clinics from February to August 2022 (08/02/2022 to 02/08/2022). At the same visit, cervical specimens were obtained for high-risk human papillomavirus (hr-HPV) DNA testing (with the Sansure MA-6000 PCR platform) followed by either visual inspection with acetic acid (VIA) or mobile colposcopy with the enhanced visual assessment system.

**Results:**

Two hundred and seventy and 107 women were screened in the antenatal and postnatal groups, respectively. The mean ages were 29.4 (SD, 5.4) in the ANC group and 28.6 (SD, 6.4) years in the PNC group. The overall hr-HPV prevalence rate was 25.5% (95% confidence interval (CI), 21.1–29.9) disaggregated as 26.7% (95% CI, 21.4–31.9) in the ANC group and 22.4% (95% CI, 14.5–30.3) in the PNC group (*p* = 0.3946). Overall, 58.9% of pregnant women (28.3% hr-HPV+) and 66.4% of postnatal women (22.5% hr-HPV+) only visited a health facility when pregnant or after delivery (at Child Welfare Clinics). The VIA ‘positivity’ rate for all screened women was 5.3% (95% CI, 3.1–7.6), disaggregated into 5.2% (95% CI, 2.5–7.8) in the ANC group and 5.7% (95% CI, 1.3–10.1) in the PNC group (*p*-value = 0.853).

**Conclusion:**

A significant number of women in Ghana only visit a health facility during pregnancy or after delivery. ANC and PNC clinics would offer opportunities to increase coverage in cervical precancer screening in low-resource settings. Relying on community nurses ensures that such programs are readily integrated into routine care of women and no opportunity is missed.

## Introduction

Despite being largely preventable, the burden of cervical cancer is heaviest in low and middle-income countries (LMICs) like Ghana where it is a leading cause of cancer-related mortality [1]. With up to 9.4 million women at risk of developing cervical cancer in Ghana, approximately 2,797 women are diagnosed with the disease yearly and 1,696 women die annually from cervical cancer [2]. In many LMICs, including Ghana, that already lack national cervical cancer screening programs, cervical screening uptake is also low, necessitating many screening approaches such as ‘screen and treat’ and ‘screen, triage and treat’ which have all been sanctioned by the World Health Organization (WHO).

The call by the WHO for the elimination of cervical cancer is premised on achieving 90% vaccination, 70% two-time screening in a lifetime, and treatment of 90% of women with cervical precancer or cancer [3]. If adhered to, the model has been predicted to drastically reduce morbidity and mortality due to cervical cancer [4]. However, in many LMICs, the three-pronged strategy is difficult to implement due to resource limitations. In the interim, some components of the strategy can be implemented until such a time that resources are available to implement all strategies within the 90:70:90 call. This approach has been predicted to also have a significant positive effect, though not comparable to the three-pronged solution, and not likely to result in the elimination of cervical cancer [4].

In Ghana, there are variations in fertility rate, with women in rural communities having almost double the fertility rate of their counterparts in urban communities resulting in a higher average number of antenatal and postnatal visits. Again, women in rural communities continue to give birth at older ages [5]. It can thus be inferred that if women routinely attend antenatal and postnatal clinics, then most women will visit the antenatal and postnatal clinics at least twice in their lifetime. Research has shown that 96% of pregnant Ghanaian women receive antenatal care (ANC) at least once, 80% attend ANC clinics at least four times, and at least 74% of rural women receive postnatal care (PNC) [6]. Pregnant women are also strongly motivated to adhere strictly to care and follow-up instructions given by healthcare workers throughout their pregnancy and soon thereafter [7]. Comparatively, the uptake of cervical precancer screening is low [8]. Better attendance at ANC and PNC clinics, as well as stricter adherence to follow-up instructions, provide an opportunity to use these visits to provide routine cervical screening services to women and thus fulfill the WHO’s call to screen at least 70% of women twice in their lifetime in efforts to eliminate cervical cancer. Women attending ANC and PNC clinics comprise a generally sexually active group, who ideally should already have been integrated into a cervical screening schedule. These clinics further provide avenues for health education which can help bridge the communication gap responsible for poor uptake of cervical screening services and knowledge about cervical cancer.

A number of previous studies have assessed the safety and implementation of cytology-based screening and high-risk human papillomavirus (hr-HPV) DNA-based testing in screening pregnant women [9–12]. Another study investigated the prevalence of HPV genotypes among pregnant Ghanaian women using vaginal swabs collected routinely prior to delivery to rule out bacterial causes of early-onset neonatal sepsis [13]. To the best of our knowledge, however, no study has investigated the prevalence of hr-HPV and preinvasive cervical lesions in the antenatal and postnatal periods.

To address this gap in the evidence, we aimed to determine the prevalence of hr-HPV infection and cervical lesions among two cohorts of women who underwent cervical precancer screening while attending ANC and PNC clinics in Battor, Ghana. Further, we discuss the feasibility of integrating cervical screening into the scope of services provided to women attending these clinics in a concerted effort to improve cervical screening coverage, particularly in rural Ghana.

## Materials and methods

### Study design, setting, and participants

This descriptive cross-sectional study was conducted to assess the prevalence of hr-HPV infection and cervical lesions among 240 pregnant women attending the ANC clinic and 94 women attending the PNC clinic after delivery at the Catholic Hospital, Battor, Ghana and those enrolled during community outreaches. The screening sessions were held on weekdays between February–August 2022 (08/02/2022 to 02/08/2022) at the Cervical Cancer Prevention and Training Centre (CCPTC) in Catholic Hospital Battor, Ghana, as well as at outreach clinics by the public health team of the hospital joined by the staff of the CCPTC.

### Sample size

We did not determine an optimal sample size before study initiation because we did not originally conduct the screening sessions within a research setting. We included women attending both antenatal and postnatal clinics who were willing and able to consent to undergo screening.

### Data collection and study outcomes

Before screening, a nurse provided each woman with detailed information on why cervical screening was necessary, what it entailed (procedure-wise), and the associated benefits and risks. After consenting verbally, the nurse collected sociodemographic, historical and clinical information with a structured questionnaire in routine use at the CCPTC. Afterward, the nurse performed cervical sampling for hr-HPV DNA testing using a dry cotton swab. A visual inspection method was done in the same setting using either a VIA or mobile colposcopy with the enhanced visual assessment (EVA system; Mobile ODT, Tel Aviv, Israel). Cervical samples were sent to the central laboratory for hr-HPV DNA testing within 7 days after collection. Information collected using the questionnaires and findings on laboratory testing and visual inspection were entered manually into REDCap version 11.0.3 (Vanderbilt University, Nashville, TN, USA) and stored in secure databases hosted at the screening centre. Prior to the analyses, the databases were queried and the data were extracted and de-identified by assigning unique alphanumeric codes to ensure privacy. The study outcomes here were either the presence of clinically-relevant lesions on visual inspection (VIA or EVA mobile colposcopy) or a positive hr-HPV DNA test.

### Cervical sample collection

Cervical sampling and visual inspection were performed by well-trained and experienced nurses at the CCPTC. In a private room, the women were placed in the dorsal lithotomy position and a sterile vaginal speculum was inserted to achieve cervical exposure. A cotton-tipped applicator was then used to gently take an ectocervical sample while taking extra precautions not to enter the cervical canal. The used applicator was then placed in a specimen collection tube, capped, and submitted to the laboratory for processing and hr-HPV DNA testing.

### VIA and EVA mobile colposcopy

After cervical sample collection, and with the woman still in the lithotomy position, the nurse applied 5% acetic acid to the cervix and inspected it with the naked eye after 2 minutes. The findings on VIA were reported as either ‘negative’ or ‘positive’ in the absence or presence of well-defined aceto-whitening at the transformation zone (TZ). EVA mobile colposcopy was performed for women with positive findings on VIA to obtain images for routine quality assurance purposes. This approach informs patient triaging and follow up. Concurrent HPV DNA testing with a visual inspection method (VIA/mobile colposcopy) obviates the need for women to be recalled for follow up when HPV DNA testing comes in as positive. This reduced loss to follow up and cost. Findings from EVA colposcopy were documented by the examining nurse using the Rio 2011 Colposcopy Nomenclature of the International Federation for Cervical Pathology and Colposcopy [14,15] as follows:

- Adequate/inadequate conditions for colposcopic assessment
- TZ type 1: squamocolumnar junction fully ectocervical; entire circumference visible
- TZ type 2: squamocolumnar junction partly or fully endocervical; entire circumference visible
- TZ type 3: squamocolumnar junction partly or fully endocervical; entire circumference not visible

#### Laboratory processing of cervical samples and testing

All cervical specimens obtained before VIA and EVA mobile colposcopy were subjected to hr-HPV DNA testing using the MA-6000 platform (Sansure Biotech Inc., Hunan, China). Sample processing and testing were performed strictly according to the manufacturer’s instructions [16], details of which have been published previously [17]. Briefly, after isolating a pure fraction of DNA in solution by adding 5 µL of the manufacturer’s sample release reagent to 5 µL of the cell suspension, it was mixed and incubated at 25°C. Following a series of 45 polymerase chain reaction (PCR) cycles, DNA amplification was performed, and fluorescence data were collected. We used the qualitative version of the MA-6000, which has been set up to identify 15 HPV types based on the detection of the following four dyes: FAM (HPV 18), CY5 (HPV 16), ROX (to collectively identify HPV 31/33/35/39/45/51/52/53/56/58/59/66/68 as hr-HPV DNA), and HEX (to detect human β-globin as an internal control). The test outputs were then read and interpreted in strict accordance with the manufacturer’s instructions.

### Ethical considerations

Ethical clearance was given by the Institutional Review Board, Research and Development Unit of the Catholic Hospital Battor, Ghana (approval no. CHB-ERC-002/07/19). Informed consent was also sought from the women prior to questionnaire administration, sample collection, and cervical screening.

### Statistical analyses

We summarise all categorical sociodemographic and clinical characteristics of the women using frequencies and proportions. Continuous variables with approximately normal distributions (e.g., age and gestational age) are summarised as means with their SDs while those with nonnormal distributions (e.g., parity) are presented as medians with their interquartile ranges (IQRs). Side-by-side box and whisker plots are used to describe the age distributions of ANC and PNC attendees by hr-HPV infection status, as well as the distribution of gestational age among ANC attendees by hr-HPV infection status. Pearson’s chi-squared test of independence was performed to explore the association between hr-HPV infection status and selected categorical sociodemographic and clinical variables. The student *t*-test was used to explore the relationship between hr-HPV positivity and selected continuous variables. The overall prevalence rates of hr-HPV infection and cervical lesions among all study participants, as well as the corresponding prevalence rates in the ANC and PNC groups, are expressed in rate form with 95% confidence intervals (CIs). The statistical analyses were performed using Stata 14.2 (StataCorp LLC, College Station, TX, USA). All tests of hypothesis were performed at a two-sided alpha level of 5%.

## Results

### Participant recruitment and selection

**Figure 1.**
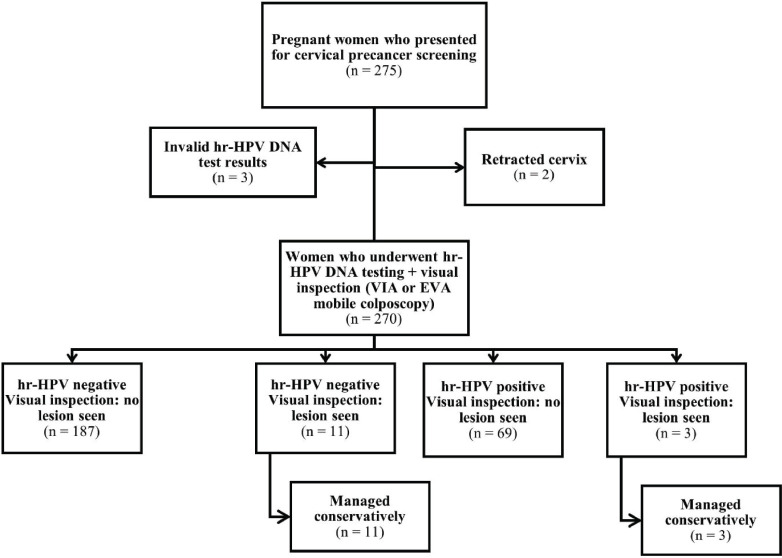
Flowchart for cervical precancer screening among women attending the ANC clinic.

**Figure 2.**
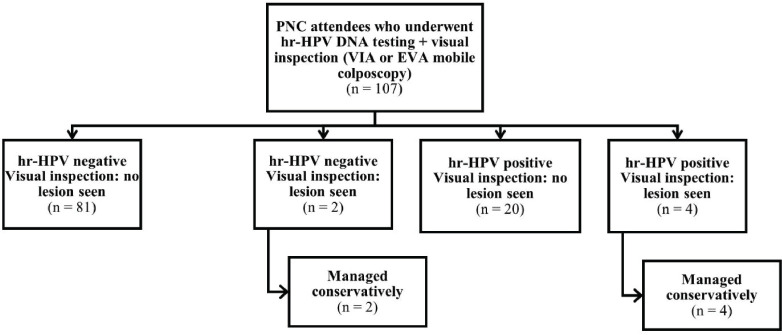
Flowchart showing outcomes of women who underwent cervical precancer screening at the PNC clinic.

Overall, 382 women (275 and 107 women attending the ANC and PNC clinics, respectively) consented to undergo screening. Three women in the ANC group who had invalid hr-HPV DNA test results and two women with retracted cervixes were excluded from the analysis (Figure 1). All 107 women attending PNC clinic who underwent screening were included in the analysis (Figure 2). Thus, a total of 377 women (*n* = 270 and 107 in the ANC and PNC groups, respectively) were included in this study.

### Sociodemographic and clinical details of the participants

The overall mean age at screening was 29.2 (SD, 5.7) years: 29.4 years (95% CI, 28.8–30.0) in the ANC group and 28.6 years (95% CI, 27.1–29.7) in the PNC group. Half of the women had at least two children and more than 70% were married. More than 75% of the women had at least a secondary school education and women attending ANC clinic were more likely to earn an income compared to their PNC counterparts (82.2% versus 68.2%). Approximately 2 out of 3 (65.1%) had a history of contraception use and 61.3% had never been to a health facility prior to the current pregnancy (58.9% versus 66.4% for the ANC and PNC groups, respectively). Overall, almost 70% of the women showed a type 3 TZ on VIA, significantly more so in the ANC group (ANC versus PNC: 75.2% versus 56.6%, *p* = 0.0004) (Table 1).

### Screening outcomes and treatment of participants

Overall, 96 of the 377 women tested hr-HPV positive, yielding an overall hr-HPV prevalence of 25.5% (95% CI, 21.1–29.9). In the ANC group, the hr-HPV prevalence was 26.7% (95% CI, 21.4–31.9) compared to 22.4% (95% CI, 14.5–30.3) in the PNC group. The VIA ‘positivity’ rate for all screened women was 5.3% (95% CI, 3.1–7.6), disaggregated into 5.2% (95% CI, 2.5–7.8) and 5.7% (95% CI, 1.3–10.1) in the ANC and PNC groups, respectively. All women who showed clinically relevant lesions on visual inspection were managed conservatively. This is in the context of the increased risk of complications that could arise because of an attempt to treat lesions during pregnancy. The hr-HPV positivity rates among women in the ANC and PNC groups who had only visited a health facility when pregnant or after delivery were 28.3% and 22.5%, respectively.

### Exploratory analysis of factors associated with hr-HPV positivity women attending ANC and PNC clinics

**Figure 3.**
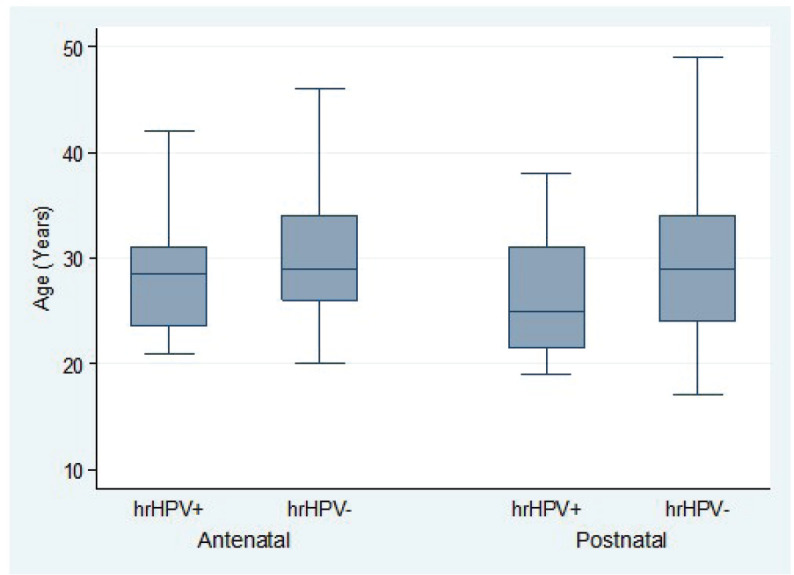
Age distribution of pregnant women attending ANC clinic and women attending PNC clinic by high-risk HPV status.

**Figure 4.**
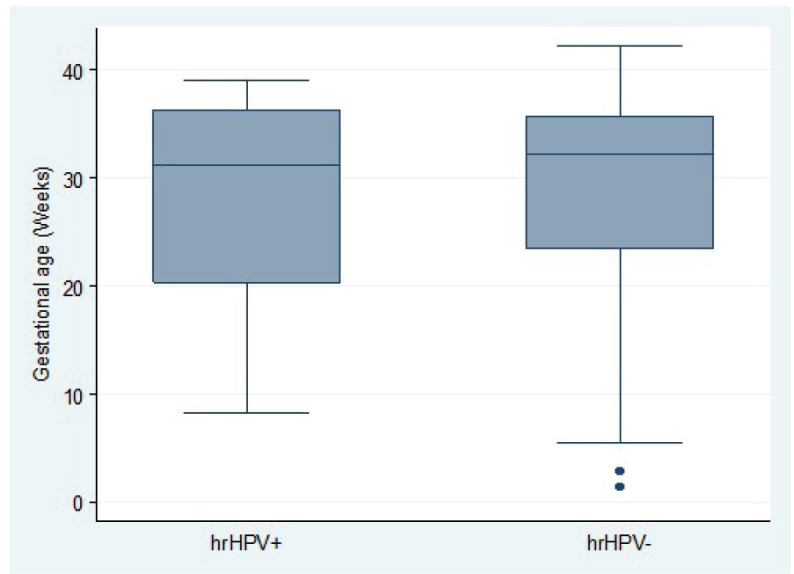
Distribution of gestational age among pregnant women attending ANC clinic by high-risk HPV status.

Overall, hr-HPV-positive women tended to be significantly younger than hr-HPV-negative women (difference in mean age: −1.9 years; 95% CI, −3.2 to −0.6; *t*-test *p* = 0.0043). This age difference stratified by hr-HPV positivity was statistically significant in the ANC group (difference in mean age: 1.7 years; 95% CI, 2.1–3.1; *t*-test *p* = 0.0250) but not statistically significant in the PNC group (difference in mean age: 2.8 years; 95% CI, −0.1 to 5.7; *t*-test *p* = 0.0588) (Figure 3). In the ANC group, there was no association between hr-HPV positivity and gestational age (difference in mean gestational age: 0.9 weeks; 95% CI, −1.6 to 3.5; *t*-test *p* = 0.4806) (Figure 4).

## Discussion

We aimed to determine the prevalence of hr-HPV infection and cervical lesions among women attending outpatient ANC and PNC clinics in Ghana who underwent cervical screening via concurrent hr-HPV DNA testing and visual inspection. We found an overall hr-HPV prevalence of 25.5%, with no statistically significant difference between the ANC and PNC groups (*p* = 0.3946). Further, 1.9% showed positive findings on both hr-HPV DNA testing and visual inspection, also with no statistically significant difference between the groups (*p* = 0.0884). Similar to the combined group, the ANC group showed a significant association between hr-HPV positivity and age; this was not so in the PNC group. There was also no significant association between gestational age and hr-HPV positivity. For those with lesions on VIA, a conservative management approach was adopted because the increased blood supply to the cervix during pregnancy puts pregnant women at risk of severe bleeding when biopsies of the cervix are taken or when treatment for cervical precancer is performed either by ablation or excision. There is also a risk of miscarriage or preterm delivery [18]. Many minor changes will regress after delivery, [19] those that persist can be treated 4–6 weeks after delivery when the risk of bleeding is less. The aim of colposcopy/screening in pregnancy is therefore to identify precancerous lesions that can be followed up and treated, if necessary, after delivery and also to identify cancerous lesions that require immediate attention. So far 4 out of the 14 antenatal clients with cervical lesions and 1 out of the 6 postnatal clients with cervical lesions have had follow up screening. The rest will be called for screening in our future training programmes. Not every woman will be lost to follow up. The Community-based Health Planning and Services (CHPS) model in Ghana uses Community Health Nurses who stay in the communities and offer preventive services. They have home visits as part of their schedule, and can follow up women in the communities. To avoid overtreatment, we prefer to manage women with minor changes (that are likely to regress spontaneously) conservatively, when the women are not likely to be lost to follow up.

The WHO in the 90-70-90 call recommends cervical cancer screening with a high-performance test (including HPV DNA testing) at 35 and 45 years. This is the minimum expected. In settings where resources are available, women can be screened earlier and more often. In fact, the WHO recommends that cervical cancer screening with HPV DNA testing should begin at the age of 30 years for the general population and 25 years, for women living with HIV. Subsequently, regular screening should be performed every 5 to 10 years for the general population and every 3 to 5 years for women living with HIV, depending on the screening method used [20]. The recommendation in many settings is to start HPV DNA testing at 30 years, but this can be done from 25 years if resources are available [21]. The age to start HPV DNA testing has been a subject of many discussions [22]. Of clinical importance are persistent HPV infections. Apart from younger women having transient HPV infections that are more likely to clear spontaneously [23] leading to overtreatment when they are screened using HPV DNA testing, this can also lead to increased workload with colposcopy in traditional screening settings using sequential HPV DNA testing with screen positives being referred for colposcopy. Our model of concurrent HPV DNA testing and a visual inspection with VIA or mobile colposcopy performed by trained nurses overcomes this challenge of lack of colposcopists and loss to follow up [24, 25, 27].

The overall and group-stratified hr-HPV prevalence rates identified here were higher than that reported by the WHO (21.3%) for women in West Africa [28, 29]. On the other hand, our estimates were lower than those reported for community-dwelling Ghanaian women in the North Tongu District (32.3%; 95% CI, 30.2–34.5) [28], as well as women seeking reproductive health care in Accra and Kumasi (35.0%; 95% CI, 29.6–40.4) [30]. The average ages of women attending the ANC and PNC clinics were 29.4 and 28.6 years, respectively, with 72 (19.1%) of women 35 years old and above, 284 (75.3%) were 25 years old and above. This is indicative of a unique cohort of young women with characteristics that may differ from the more heterogeneous group of women who would usually participate in general screening programs. In both groups, the median parity was two, suggesting that there is a possibility of repeat ANC and PNC visits in a majority of these women, allowing them to be screened at least twice in a lifetime if screening is initiated at the first ANC or PNC visit. In this age category, a highly sensitive test such as hr-HPV DNA test is needed to determine the presence of infection that may persist and lead to cervical cancer and may only be picked up during these clinics for follow-up. In pregnancy and the weeks following delivery, there is an increase in cervical volume and eversion of the endocervical columnar epithelium unto the ectocervix, resulting in active squamous metaplasia [31]. This makes it easier to visualise the squamocolumnar junction. On the other hand, this squamous metaplasia is associated with VIA or EVA mosaic or punctate patterns [32], making it difficult to differentiate it from low-grade squamous intraepithelial lesion on screening. The decision to use hr-HPV DNA screening is further supported by the high rates of TZ type 3 (70%) on VIA. Without adequate visualisation of the full TZ, lesions may be missed if a visual inspection method is solely relied upon to screen such women. A hr-HPV-based approach to triaging is also more attractive because hormonal influences of pregnancy on the endocervical glands increase mucus production, obscuring cervical visualisation [33].

The absence of adequate cytologists/cytotechnologists and infrastructure for running national cytology-based cervical cancer screening programs in LMICs makes it important to use approaches that can be decentralised to reach more women. Point-of-care molecular tests (including HPV tests) as well as the increased availability of PCR platforms which can be used for HPV testing in LMICs, offer the opportunity for women to be screened in antenatal and postnatal clinics in decentralised settings like the CHPS compounds in Ghana where Community Health Nurses and Midwives offer antenatal and postnatal services [26]. The high sensitivity and negative predictive value of HPV DNA testing means women with a negative HPV test results have low probability of developing cervical lesions in the next 5 years. The next screening, therefore, can be scheduled for 5 to 10 years. This will make it possible to concentrate resources on the smaller proportion of women who test positive for high-risk HPV. Decentralised screening by health workers who stay in the communities with these women, and even offer home visits, means a ‘see and treat approach’ does not always have to be done, even in the postnatal period, because there is less risk of loss to follow up. Treatment can be restricted to the women who really need it, to save resources [27].

Although there is a fear of pregnancy loss with cervical precancer screening [34], we did not experience this in our study. In fact, one woman (23-year-old; estimated gestational age, 30 weeks) was found to have bulging membranes through the endocervical canal. She underwent an emergency cerclage and was delivered via a cesarean section that resulted in a healthy live twin birth at 38 weeks and 1 day. Without the screening, this could have resulted in a miscarriage/preterm delivery. Cervical precancer screening in pregnancy also offers an opportunity to pick up and treat vaginal infections which could lead to miscarriages and preterm deliveries.

With no national cervical cancer screening program in place in Ghana it is important that every visit by a woman to the hospital is seen as an opportunity for cervical cancer screening. Given that approximately 60% of women aged ≥25 years achieve adequate ANC attendance (4–7 visits) [35] and that these women fall within the recommended age group for cervical cancer screening [36, 37], it may be innovative and acceptable to incorporate hr-HPV DNA testing into routine ANC care in attempts to increase coverage among women of reproductive age. This ties in with the WHO’s strategy to achieve 70% screening target in their 90:70:90 strategy , as spelt out in the strategic document [38]. Also, during these visits, education on cervical cancer prevention methods such as HPV vaccination can be given to ensure that women are aware of the options available to them. This approach could be particularly useful in settings with a high prevalence of HIV as women living with HIV are six times more likely to develop cervical cancer compared to women without HIV, HPV infections are less likely to be cleared spontaneously, precancerous lesions of the cervix progress faster to cancer, requiring that these women are screened more often than the general population [39].

The decision to participate in cervical screening while receiving ANC or PNC would likely be guided by an interplay between individual and social factors that are best elucidated via qualitative research. In doing so, specific attitudes and beliefs of women can be targets for change in improving cervical screening uptake in ANC and PNC clinics. The current low levels of awareness of cervical cancer [8] together with the increasing prevalence of HIV in Ghana [40] may hinder efforts aimed at reducing cervical cancer incidence. This is further worsened by the lack of a comprehensive national cervical cancer screening policy that specifically targets special populations of women who seek healthcare for other reasons, such as pregnant women and those presenting for postnatal assessment. There is a need for concerted efforts from all levels of government at the national and sub-national levels, with an interim intervention being persistent education at ANC and PNC clinics, with the ultimate aim of making hr-HPV-based cervical screening a key component of infectious disease screening in the antenatal and postnatal periods.

## Limitations and strengths

This study had some limitations. First, due to resource limitations, we used the qualitative version of the MA-6000 test and did not perform full genotyping for hr-HPV-positive women. We could therefore neither determine the exact HPV types among the women screened nor identify coinfections. Second, having drawn participants conveniently from women who were attending ANC and PNC clinics at our hospital, the study samples may not represent all women attending ANC and PNC clinics in Ghana. Further, we did not prospectively follow up hr-HPV-positive women to differentiate between transient and persistent infections. The strength of this study is in its rural setting. Successful implementation of such programs is crucial for the success of cervical cancer screening programs in low resource settings. Again, the reliance on nurses in the community ensures that services are available and integrated into routine care of women.

## Conclusion

The opportunity and convenience of performing cervical screening among women attending ANC and PNC clinics may contribute significantly to increasing coverage and reducing the incidence of cervical cancer and its associated morbidity and mortality in Ghana. Including such an approach in a national guideline would help ensure that practitioners do not miss the detection of identifiable precancerous and cancerous lesions among women that are lost to follow-up upon exiting the healthcare system after completing their antenatal and postnatal assessments.

## Conflicts of interest

The authors declare that they have no conflict of interest.

## Notation of prior abstract presentation

This work has been partially presented as an abstract poster at the 35th International Papillomavirus Conference in Washington DC. from April 17 to 21, 2023.

## Funding

This project was funded by the Cervical Cancer Prevention and Training Centre, Battor, Volta Region, Ghana.

## Author contributions

Conceptualisation and study design: KE, ET, CMW, GBK, BHA, LSM and JEA. Screening and data collection: GBK, CMW, ET, HTB, SK, SD and KE. Data management and formal analysis: JEA, SD, ET, CMW, NOE and KE. Writing – original draft: PKA, NOE, JEA, HTB, ET and KE. All the authors read and approved the manuscript in its current form.

## Figures and Tables

hr-HPV, high-risk human papillomavirus; VIA, visual inspection with acetic acid

hr-HPV, high-risk human papillomavirus; VIA, visual inspection with acetic acid; PNC, postnatal care

**Table 1.** Distributions of sociodemographic and clinical characteristics among women receiving antenatal and PNC who underwent cervical precancer screening via hr-HPV DNA testing and visual inspection (VIA or EVA mobile colposcopy).

Characteristic	Antenatal (*n* = 270)	Postnatal (*n* = 107)	Overall (*n* = 377)
Age, mean (SD)	29.4 (5.4)	28.6 (6.4)	29.2 (5.7)
Marital status, % (*n*) Single Has a steady partner Married	1.1 (3) 28.5 (77) 70.4 (190)	0.9 (1) 28.0 (30) 71.0 (76)	1.1 (4) 28.4 (107) 70.6 (266)
Parity, median (IQR)	2 (0, 3)	2 (1, 4)	2 (1, 3)
Education level, % (*n*) No formal education Elementary Secondary Tertiary Vocational/technical/other	7.8 (21) 15.2 (41) 64.8 (175) 11.9 (32) 0.4 (1)	4.7 (5) 22.4 (24) 65.4 (70) 6.5 (7) 0.9 (1)	6.9 (26) 17.2 (65) 65.0 (245) 10.3 (39) 0.5 (2)
Religion, % (*n*) Christian Islam African traditional religion Other	95.9 (259) 2.2 (6) 0.7 (2) 1.1 (3)	98.1 (105) 0.9 (1) 0.0 (0) 0.9 (1)	96.6 (364) 1.9 (7) 0.5 (2) 1.1 (4)
Past contraceptive use, % (*n*)	64.4 (174)	68.2 (73)	65.5 (247)
Prior cervical screening, % (*n*)	4.8 (13)	3.7 (4)	4.5 (17)
Only visited a health facility when pregnant or after delivery, % (*n*)	58.9 (159)	66.4 (71)	61.0 (230)
Earns income, % (*n*)	82.2 (222)	68.2 (73)	78.2 (295)
Smoker, % (*n*)	0.0 (0)	0.0 (0)	0.0 (0)
HIV status, % (*n*) Positive Negative Unknown	1.5 (4) 68.9 (186) 29.6 (80)	1.9 (2) 50.5 (54) 47.7 (51)	1.6 (6) 63.7 (240) 34.7 (131)
Gestational age, weeks; mean (SD)^§^	28.9 (8.7)	-	-
No. of weeks post-delivery, % (*n*) ≤5 6 7 8 ≥9 Missing	- - - - - -	8.4 (9) 49.5 (53) 17.8 (19) 15.0 (16) 8.4 (9) 0.9 (1)	- - - - - -
TZ type on VIA*, % (*n*) 1 2 3	9.3 (25) 15.6 (42) 75.2 (203)	16.0 (17) 27.4 (29) 56.6 (60)	11.2 (42) 18.9 (71) 69.9 (263)
TZ type on EVA**, % (*n*) 1 2 3	0.0 (0) 6.7 (1) 93.3 (14)	- - -	- - -
EVA positive, % (95% CI)**	80.0 (59.8–100.0)	-	-
VIA positive, % (95% CI)*	5.2 (2.5–7.8)	5.7 (1.3–10.1)	5.3 (3.1–7.6)
hr-HPV positive, % (95% CI)	26.7 (21.4–31.9)	22.4 (14.5–30.3)	25.5 (21.1–29.9)

^§^Data on gestational age available for 226 women; *One woman underwent EVA colposcopy but not VIA; **15 women were screened via EVA mobile colposcopy

TZ, transformation zone; VIA, visual inspection with acetic acid; HIV, human immunodeficiency virus; SD, standard deviation; IQR, interquartile range; hr-HPV, high-risk human papillomavirus
